# Are the Capillaries of the Enamel Capable of Sustaining Metabolism?

**Published:** 1914-04-15

**Authors:** Theo. Von Beust

**Affiliations:** Dresden, Germany


### ARE THE CAPILLARIES OF THE ENAMEL CAPABLE OF SUSTAINING METABOLISM?

BY THEO. VON BEUST, D.D.S., M.D., DRESDEN, GERMANY.

In vol. lv, No. 10 (October 1913), of the Dental Cosmos, Prof. H. P. Pickerill questions the merit of the conclusions expressed in an article entitled "A Contribution to the Study of Immunity to Dental Caries," which appeared in vol. liv, No. 6 (June 1912) of this journal.

In his highly interesting article, Professor Pickerill exhibits three figures (Figs. 37, 38 and 39) in which the tissues had been stained by my method of capillary attraction. These serve to illustrate the view expressed under "(8)" in the summary of his contribution, which reads: "There is not sufficient evidence to show that normal secretions or bodily juices may pass from the dentin to the enamel."

The photographs shown force me to conclude that Professor Pickerill expected to find a general staining of the tissue of the enamel rods, which alone would result in the diffuse staining which he was evidently expecting and which failed to appear in his preparations. As a matter of fact, the enamel of the specimens prepared in the manner described by me in the above-mentioned article (ground in paraffin oil and mounted in balsam) as a rule appears, when examined microscopically or when enlarged to the size of the cuts shown by Dr. Pickerill, perfectly white. If sufficiently magnified to disclose the enamel capillaries, however, it will be seen that all the vessels pictured by me in the Dental Cosmos have absorbed and transmitted the stain. These findings establish beyond a doubt the existence of a communication between the pulp and the surface of the enamel by way of the dentinal tubes and the canals of the enamel, and lead me to conclude that metabolism is possible, and probable.

In the report cited by Professor Pickerill I made no allusion whatever to a staining of the enamel rods. That they occasionally do stain, as Professor Pickerill declares—and, I may add, very frequently in recently erupted teeth—only serves to substantiate my claim that tissue changes take place in the enamel of the erupted tooth. It was no "very occasional result" upon which I based my ideas. On the contrary, in no instance have I been unsuccessful in finding the enamel capillaries stained, and the number of teeth examined is by no means small. I venture to say that the stain in the enamel vessels in the very specimens exhibited by Professor Pickerill can be demonstrated, provided that they were ground in oil of a neutral reaction.

Professor Pickerill's objection to alcoholic stains, owing to their volatility, is worthy of mention, but it happens to have no practical bearing upon the subject. The enamel capillaries will convey watery stains also. (See *Archiv fur Zahnheilkunde* for November 1913.) Too little is known, furthermore, of the relative diffusibility of the protein derivatives and salts entertaining tissue metabolism, as compared to coloring matters, to admit of any conclusions in this regard.

Nitrate of silver will penetrate the enamel vessels in the manner described by me, about as well as any other stain. Proof: The root of a freshly extracted tooth is sawed through about midway between the apex and the crown, and the root and pulp cavity enlarged to receive the end of a glass funnel or pipet. The surface of the root is superficially ground (up to the enamel line) and the sawed end and ground surface shellacked, care being taken to prevent the varnish from reaching the inner part of the pulp cavity or surface of the crown. The funnel, containing a wire, is now sealed into the tooth with sticky-wax, allowing the wax to spread over the shellacked part of the tooth. After the wax has cooled, a few drops of water are placed into the funnel and the wire withdrawn (to exclude the air!). One per cent. nitrate of silver in aqueous solution is now added and the specimen is placed in the dark. After the lapse of a few days the teeth are ground in paraffin oil, the sections are wiped and placed in a bottle containing dilute alcohol. The bottle is now placed in the sun until decomposition of the salt has taken place, whereupon the specimens are passed through strong and finally through absolute alcohol (to extract the water), washed in xylol, and imbedded in balsam. Microscopical examination discloses the fact that the silver solution has followed the dentinal tubes and permeated all uncalcified tissue of the enamel, *i.e.* the capillary spaces.

The cross section of the enamel fibers displaying the arrangement of the capillaries, which Professor Pickerill shows in his Fig. 12 and Fig. 33, resembles my own illustration Fig. 3. These canals are in my opinion identical with the sinuosities to which the name spindles and tufts have been given. They certainly stand in direct communication with the dentinal tubes. My experiments verify the view expressed by Professor Pickerill that these "interprismatic spaces" are continuous, and show, moreover, that all other spaces in the enamel are parts of one great system. That their origination is the result of "imperfectly acquired function," however, I do not believe. These canals are found in all teeth, including the teeth of the anthropoid apes raised in their native habitat. Their occurrence is so general and their morphological uniformity so constant, that anyone about to grind a specimen can as logically expect to find the characteristic appearances produced by the presence of these capillaries, as an accoucheur would expect to find fingers and toes on a babe being born.—*Dental Cosmos.*

